# Plants regulate the effects of experimental warming on the soil microbial community in an alpine scrub ecosystem

**DOI:** 10.1371/journal.pone.0195079

**Published:** 2018-04-18

**Authors:** Zhiliang Ma, Wenqiang Zhao, Chunzhang Zhao, Dong Wang, Mei Liu, Dandan Li, Qing Liu

**Affiliations:** 1 Key Laboratory of Mountain Ecological Restoration and Bioresource Utilization & Ecological Restoration Biodiversity Conservation, Key Laboratory of Sichuan Province, Chengdu Institute of Biology, Chinese Academy of Science, Chengdu, China; 2 College of Life Sciences, Sichuan University, Chengdu, China; 3 University of Chinese Academy of Sciences, Beijing, China; University of Massachusetts, UNITED STATES

## Abstract

Information on how soil microbial communities respond to warming is still scarce for alpine scrub ecosystems. We conducted a field experiment with two plant treatments (plant removal or undisturbed) subjected to warmed or unwarmed conditions to examine the effects of warming and plant removal on soil microbial community structures during the growing season in a *Sibiraea angustata* scrubland of the eastern Qinghai–Tibetan Plateau. The results indicate that experimental warming significantly influenced soil microbial biomass carbon (MBC) and microbial biomass nitrogen (MBN), but the warming effects were dependent on the plant treatments and sampling seasons. In the plant-removal plots, warming did not affect most of the microbial variables, while in the undisturbed plots, warming significantly increased the abundances of actinomycete and Gram-positive bacterial groups during the mid-growing season (July), but it did not affect the fungi groups. Plant removal significantly reduced fungal abundance throughout the growing season and significantly altered the soil microbial community structure in July. The interaction between warming and plant removal significantly influenced the soil MBC and MBN and the abundances of total microbes, bacteria and actinomycete throughout the growing season. Experimental warming significantly reduced the abundance of rare taxa, while the interaction between warming and plant removal tended to have strong effects on the abundant taxa. These findings suggest that the responses of soil microbial communities to warming are regulated by plant communities. These results provide new insights into how soil microbial community structure responds to climatic warming in alpine scrub ecosystems.

## Introduction

Increasing concentrations of atmospheric greenhouse gases are expected to raise the global mean surface temperature by 2°C to 4.5°C above pre-industrial levels by 2100, and the warming effect will be especially notable in high-altitude regions [1]. Warmer soil temperatures caused by increased air temperature could greatly influence the carbon (C) and nutrient cycling in terrestrial ecosystems [2], affecting characteristics like soil respiration, soil organic matter decomposition and soil microbial community structure and activity [3,4]. Previous studies have reported that belowground responses to warming, especially for the soil microbial community, differ greatly among ecosystem types [4,5]. Thus, a better understanding of how the soil microbial community responds to soil warming would facilitate the understanding of C and nutrient cycling in terrestrial ecosystems.

Soil microbial communities are important components of the belowground ecosystem and drive soil biogeochemical cycling, which influences terrestrial ecosystem functions [6]. Warming can alter biotic and abiotic factors, such as the quality and quantity of litter inputs, the plant community and soil moisture and nutrients, which regulate soil microbial community structures and activities and related microbial processes through both direct and indirect mechanisms [7,8]. However, there is no consistent pattern of the effects of warming on soil microbial communities, and the results of studies mainly depend on the experimental warming approach, duration and range of the warming treatment, and ecosystem type [9,10]. Moreover, warming can alter the patterns of soil microbial substrate use and directly influence soil microbial community structure [11]. Different soil microbial groups have different sensitivities to environmental changes caused by warming, such as changes in C and nutrient availability. The positive, negative or neutral effects of warming on soil microbial communities have been observed in many ecosystems [12,13,14]. For example, due to changes in soil properties and the plant community, a short-term warming directly resulted in a rapid shift in the soil microbial community structure, with a significant increase in the abundances of actinomycete biomarkers and a decrease in fungal biomarkers, in an alpine timberline zone on the eastern Qinghai–Tibetan Plateau [15]. In a tallgrass prairie ecosystem, experimental warming significantly enhanced fungal biomarkers but reduced bacterial biomarkers. As a result, the ratio of fungi to bacteria increased by 22%-63% in warmed plots, mainly due to increases in plant growth and decreases in soil nitrogen (N) and moisture availability [16]. However, contrasting warming effects on the soil microbial community structure were also observed by Rinnan et al. (2007), who reported that warming significantly decreased the ratio of fungi to bacteria in a subarctic heath ecosystem, mainly because of warming-induced changes in the plant biomass and plant community composition [17]. These results indicated that warming combined with plant community characteristics can potentially have both direct and indirect impacts on soil microbial communities. However, the direction and magnitude of the combinative effects of warming and plant community on soil microbial communities remain uncertain. We still lack a clear understanding of how soil microbial community structures respond to warming and how shifts in the soil microbial community structure influence soil C and nutrient cycling.

The alpine scrub ecosystem distributed on the Qinghai–Tibetan Plateau is among the most sensitive regions to climatic warming [1]. Occupying a total area of 1.06 × 10^5^ km^2^, it is widely distributed across the transition zones between the alpine forest and grassland [18]. These alpine ecosystems play important roles in regulating C and nutrient cycling both regionally and globally [19]. Compared with the alpine forests and grasslands on the Qinghai–Tibetan Plateau, the alpine scrub ecosystem has environmental conditions (e.g., soil moisture and temperature) that are more severe for soil microbial activity, due to lower shrub species diversity and shrub vegetation coverage and higher day-night temperature difference. Hence, the responses of the soil microbial community to warming in these alpine scrub ecosystems might be different than those in the alpine forest and grassland ecosystems, and the effects of climatic warming on soil microbial processes in these alpine regions are greatly uncertain. Previous studies that have examined the effects of environmental changes on the alpine scrub ecosystems of the Qinghai–Tibetan Plateau mainly focused on the effects of N addition on ecosystem C and N pools [18] and soil C fluxes [20]. So far, the responses of the soil microbial community structure and diversity to warming in these Qinghai–Tibetan Plateau alpine scrub ecosystems have received little attention. Hence, more information about the effects of warming on soil microbial community structure is urgently needed for the prediction of future changes in the soil microbial community and soil C and nutrient cycling in these alpine scrub ecosystems under climatic warming.

In addition, intense disturbances caused by human activities, such as over-grazing and harvesting shrubs for fuel, are dramatically changing the scrubland vegetation coverage and soil processes in these alpine scrub regions. Changes in vegetation coverage alter the soil microenvironment (e.g., soil moisture and nutrient) and thus indirectly influence the soil microbial community and its related microbial process [21]. Moreover, the plant community supplies available C and nutrient sources for soil microbes through litter inputs and root exudates, which plays an important role in shaping the soil microbial community [22]. In general, warming could strongly promote plant growth in alpine ecosystems and improve soil nutrient conditions, which could in turn increase soil microbial biomass and activity [23]. However, the increase in plant productivity caused by warming might trigger nutrient limitation for soil microbes by enhancing the competition for soil nutrients between plant communities and soil microbes, which might in turn reduce the soil microbial biomass [24]. Warming-induced changes in plant properties were found to explain ~20% of the microbial community variance in an alpine meadow on the Qinghai–Tibetan Plateau [25]. Therefore, it is expected that warming and plant communities may interactively influence soil microbial communities in alpine scrub ecosystems on the Qinghai–Tibetan Plateau. However, previous investigations on the interactive effects of warming and change in vegetation coverage on soil microbial communities have focused on grassland ecosystems [16,25], while few studies have been conducted in alpine scrub ecosystems. This has limited the understanding of the effects of warming and vegetation coverage changes on soil microbial communities and related soil microbial processes in these alpine scrub ecosystems.

Hence, in this study, we designed a field experiment including two plant treatments (plant removal or undisturbed) subjected to two temperature conditions (warmed or unwarmed) in *Sibiraea angustata* alpine scrubland. *Sibiraea angustata*, an endemic alpine shrub species, is widespread over the eastern margin of the Qinghai-Tibetan Plateau [18]. Our objectives are to elucidate how soil microbial biomass and community structure respond to short-term warming in the alpine scrub ecosystem. We hypothesized that (1) different taxa of soil microbes would exhibit different responses to the experimental warming, due to differences in temperature sensitivities and a diversified pattern of substrate use, and (2) the effects of soil warming on the soil microbial community would be mediated by plant treatments.

## Materials and methods

### Site description

The study was conducted at the Songpan Plateau Scrub Ecosystem Research Station of the Chinese Academy of Science, Sichuan Province, China (32°59’ N, 103°40’ E). The study site is located at an elevation of 3300 m and has a typical alpine climate. The mean annual temperature is approximately 4.8°C, and the mean annual precipitation is 693.2 mm, with over 80% concentrated in the period from May to August. The soil freezing duration is from November to April. The soil is classified as Cambisol. The pH, bulk density, soil organic carbon, and total nitrogen in the surface soil (0–15 cm) are 5.79, 0.67 g cm^-3^, 89.24 g kg^-1^, and 7.73 g kg^-1^, respectively. At this study site, the plant community was dominated by temperate and cold-temperate vegetation. *S*. *angustata*, *Salix oritrepha*, *Spiraea alpina* and *Potentilla fruticosa* were the dominant shrub species. The grass layer was dominated by *Festuca ovina*, *Gentiana striata*, *Deyeuxia flavens* and *Primula sikkimensis*.

### Experimental design and treatments

Based on our previous field investigations, three replicate sampling sites of 20 × 20 m were established in a flat stand with similar microhabitat characteristics (plant species and community structures) in early October 2015. The three replicate sampling sites were separated from one another by approximately 10 m. We used the randomized block design in which the two plant treatments (plant removal or undisturbed) were randomly set up in 5 × 5 m blocks at each sampling site. At the plant-removal plots, we removed all the litter and the aboveground biomass of shrub and grass species. To avoid the disturbance effects from the neighboring plants, we excavated a trench of 0.2 m wide and 0.75 m deep (below which few roots existed [18]). After lining the trench with a 2 mm thick plastic sheet (compound crystal film), the trench was refilled with soil to prevent adjacent plant roots from growing into the plots. The plant-removal plots were then kept free of seedlings and herbaceous vegetation by periodical manual removal throughout the experiment period. At the undisturbed plots, we did not take any measures to maintain the naturally growing shrub and grass.

To increase the soil temperature, an open top chamber (OTC) was set up in each warming plot. Based on the height and coverage of the scrub community, the OTC used in this study was quadrangular, 160 cm high, and made of high solar transmittance material with an area of 2.56 m^2^. In the undisturbed plots, *S*. *angustata* grew in the center of the OTCs. Control plots (unwarmed) were also established for the two plant treatments in each sampling site. The OTC installations were completed in late October 2015, and observations were initiated from late April 2016, providing an equilibrium period of 6 months to minimize disturbance effects. The following four treatments were used in this study: (1) plant removal + unwarmed (P0W0), (2) plant removal + warmed (P0W1), (3) undisturbed + unwarmed (P1W0), and (4) undisturbed + warmed (P1W1).

### Microclimates monitoring

To quantify the environmental factors affected by the OTCs, automatic recording systems were set up in the experimental plots. Air temperature at 70 cm high and soil temperature at 5 cm depth in the center of the plots were measured by the sensors (DS1921G-F5, Maxim Integrated Products, Dallas Semiconductor Inc., Sunnyvale, CA, USA), which were connected to a datalogger (Campbell AR5, Avalon, USA). Data were taken at 120 min intervals with the automatic recording system from early May to late October in 2016. Soil volumetric moisture at 5 cm depth was measured with a hand-held probe (IMKO, Germany) at a 1-month interval.

### Soil sampling

Soil samples were collected from the topsoil (0–15 cm) in the early (20 May), mid- (21 July), and late (21 September) growing season of 2016. Five soil cores (3 cm in diameter) were randomly taken at each plot. These five soil cores from each plot were combined to form one composite sample. The surface litter was carefully removed before the soil cores were collected. Samples were immediately transported to the laboratory in insulated boxes. After removal of the visible living plant materials, each composite sample was passed through a 2 mm sieve. Then, each composite soil sample was divided into two subsamples. One subsample was immediately used to determine the soil water content, and the other was kept in the refrigerator at 4°C and processed within two weeks to determine soil microbial biomass C (MBC), soil microbial biomass N (MBN), and microbial community structure.

### Soil analysis

The soil water content, as a conversion factor between the fresh and dry soil, was measured by oven-drying the soil for 48 h at 105°C. MBC and MBN were determined using the chloroform fumigation-extraction method [26]. Soil extractable organic C and total N in the K_2_SO_4_ extracts before and after the fumigation were quantified using a total C/N analyzer (Multi-N/C 2100, Analytik Jena AG, Germany). The released C and N were converted to MBC and MBN, respectively, using *k*_*EC*_: 0.45 and *k*_*EN*:_ 0.45 [27]. Phospholipid fatty acids (PLFAs) were extracted from 8 g of fresh soil and analyzed according to Bossio and Scow (1998) [28]. Concentrations of each PLFA were calculated relative to methyl nonadecanoate (19:0) internal standard concentrations. Total microbial PLFAs were considered to be represented by all the detected PLFAs. Bacterial biomarkers were considered to be represented by the 9 PLFAs (i15:0, a15:0, i16:0, i17:0, a17:0, 16:1ω7, 18:1ω7, cy17:0 and cy19:0 ω8) [29,30,31]. Terminally branched PLFAs, i.e., i15:0, a15:0, i16:0, i17:0, and a17:0, were used as indicators of Gram-positive bacteria (G+), whereas cy17:0, cy19:0 ω8, 16:1ω7c, and 18:1ω7, were used as indicators of Gram-negative bacteria (G-) [29,30]. Fungal biomarkers were considered to be represented by the PLFA 18:2ω6,9, and actinomycete biomarkers were considered to be represented by the 3 PLFAs (10 Me 16:0, 10 Me 17:0, and 10 Me 18:0) [29,30,31]. In addition, other PLFAs such as 14:0, 15:0, 17:0 and 18:0 were also to assess the variability of the soil microbial community composition.

We introduced biodiversity indices such as the Shannon-Wiener diversity index, Margalef richness index, Pielou evenness index, and Simpson dominance index to analyze the microbial PLFA diversities [30]. The soil microbial PLFA diversity indices were calculated using the following equations:

$$\text{Shannon-Wiener diversity index}\;(H):\;H=-\sum_{}^{}P_{i}lnP_{i}$$ (1)

Margalef richness index(R):R=(S-1)/lnN (2)

Pielou evenness index(E):E=H/lnS (3)

$$\text{Simpson dominance index}\;(C):\;C\text{=}\sum P_{i}^{2}$$ (4)

In which *P*_*i*_ is the proportional abundance of a given PLFA, *S* is the number of total PLFAs, and *N* is the sum of total PLFAs.

### Statistical analyses

Soil microbial community composition was analyzed by a principal component analysis (PCA) of the relative content of individual fatty acids for each treatment. A repeated measures ANOVA was used to assess the effects of experimental warming, plant treatment, sampling date, and their interactions on soil microbial variables. A mixed model was used to examine the effects of warming, plant removal and their interaction on soil microbial variables at each sampling date. Because of our interests in warming effects mediated by plant treatment, Student’s t-tests were performed to assess the effects of warming on each microbial variable at a given plant treatment and sampling date. Likewise, Student’s t-tests were also used to compare the differences in the daily mean soil and air temperatures and the soil volumetric moisture between the warmed and unwarmed plots at a given plant treatment. The statistical tests were considered significant at the *P* < 0.05 level. All statistical tests were performed using SPSS (version 20.0 for windows, IBM, Armonk, New York, USA).

## Results

### Microclimates

**Fig 1:**
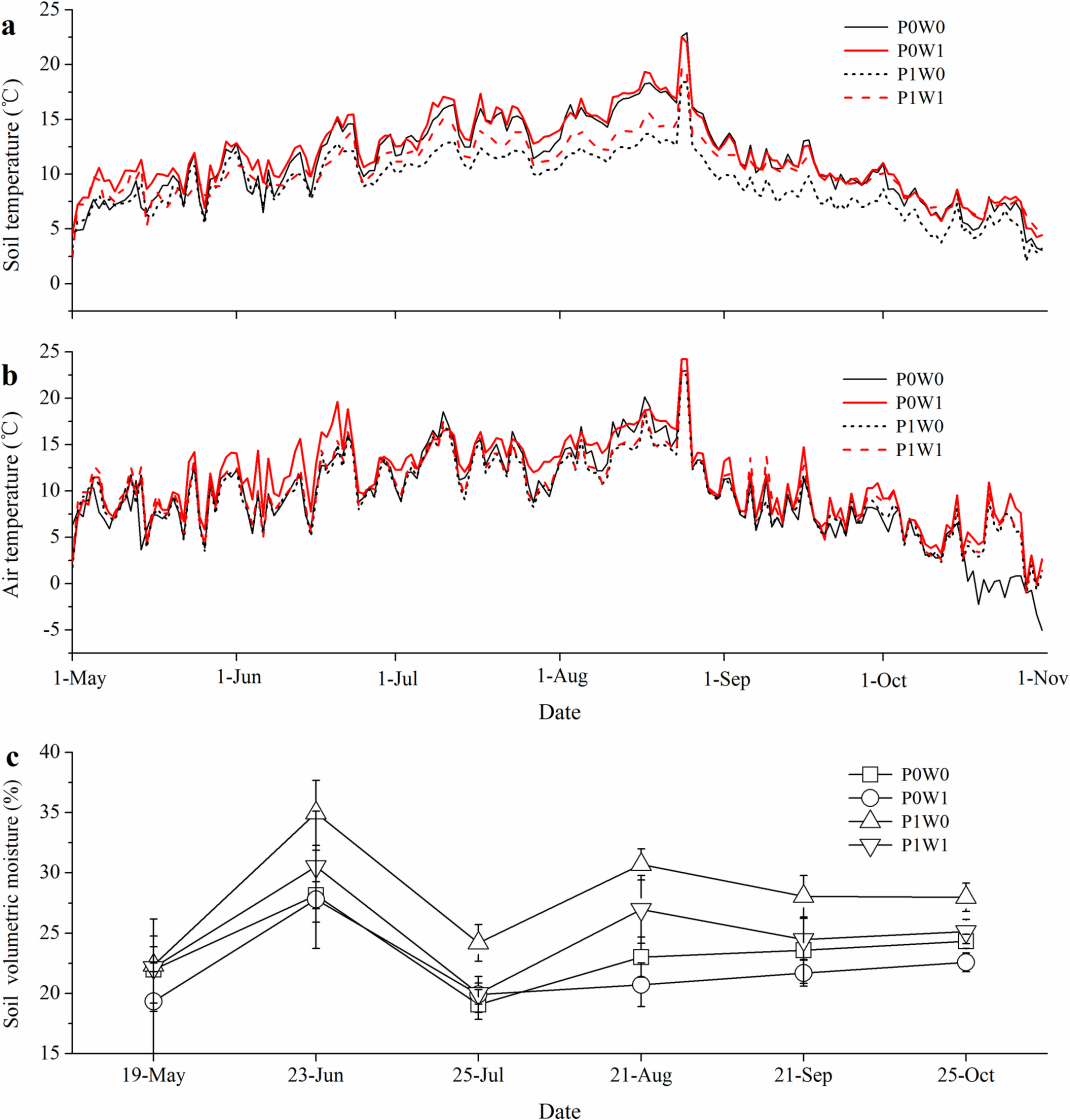
**Seasonal transitions of daily mean soil temperature at 5 cm depth (a), daily mean air temperature at 70 cm high (b) and soil volumetric moisture at 5 cm depth (c) in warmed and unwarmed plots.** P0W0, P0W1, P1W0, P1W1 refer to the treatments, indicating plant removal + unwarmed, plant removal + warmed, undisturbed + unwarmed, undisturbed + warmed, respectively.

The OTCs did cause expected warming effects within the experimental plots. Compared with the unwarmed plots, the soil temperature and air temperature in the OTCs of the plant-removal plots were on average increased by 0.64°C and 1.64°C (*P* < 0.05), respectively, while those of undisturbed plots were increased by 1.29°C (*P* < 0.05) and by 0.53°C, respectively, throughout the experimental periods (Fig 1A and 1B). Warming produced significant declines in soil volumetric moisture at 5 cm depth. Soil volumetric moisture in the warmed plots were on average 1.35% and 3.17% lower than those in the unwarmed plots in the plant-removal plots and undisturbed plots, respectively (Fig 1C).

### Soil MBC and MBN

**Fig 2:**
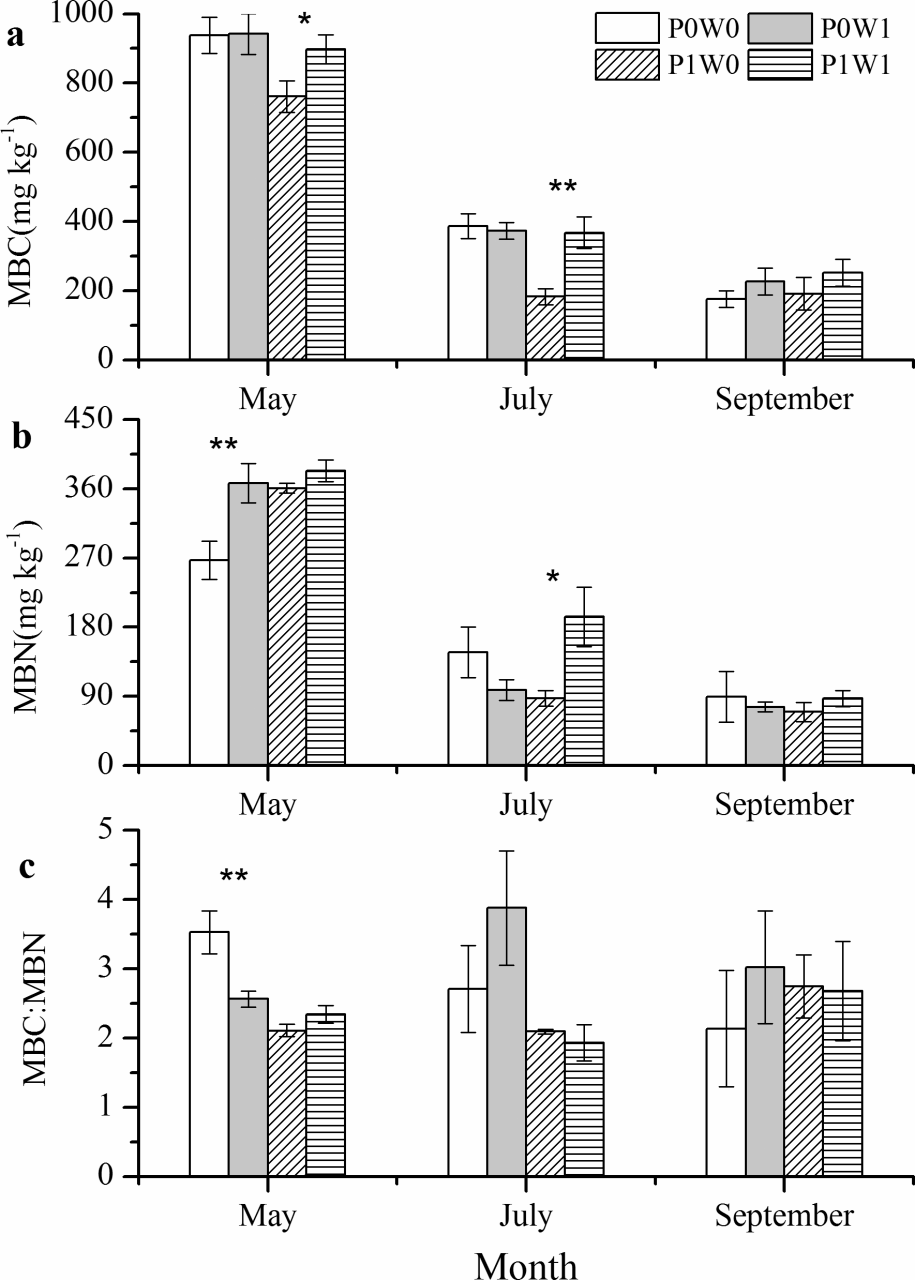
**Effects of experimental warming on soil MBC (a), MBN (b) and the ratio of MBC to MBN (MBC:MBN, c) at each sampling date.** P0W0, P0W1, P1W0, P1W1 refer to the treatments, indicating plant removal + unwarmed, plant removal + warmed, undisturbed + unwarmed, undisturbed + warmed, respectively. Asterisks indicate there were significant differences between the warmed and unwarmed plots at a given plant treatment (plant removal or undisturbed). * *P* < 0.05, ** *P* < 0.01.

Experimental warming significantly (*P* < 0.05) increased soil MBC in both May and July in the undisturbed plots, whereas no significant warming effect was found in the plant-removal plots (Fig 2A, S1 Table). For soil MBN, warming did cause significant (*P* < 0.05) increases in the plant-removal plots in May and the undisturbed plots in July (Fig 2B). However, warming only significantly (*P* < 0.05) decreased the ratios of MBC to MBN (MBC:MBN) in May in the plant-removal plots (Fig 2C and Table 1). In addition, soil MBC and MBN significantly (*P* < 0.05) varied among sampling dates (Table 1), with higher values in May and a gradual reduction in July and September (Fig 2A and 2B). Moreover, there were significant (*P* < 0.05) effects of plant removal on soil MBC, MBN and MBC:MBN, and there were significant (*P* < 0.05) interaction effects of warming and plant removal on soil MBC and MBN (Table 1).

**Table 1:** Results of the repeated measures ANOVA showing the *P* values for the responses of MBC (mg kg^-1^ dry soil), MBN (mg kg^-1^ dry soil), MBC:MBN, and PLFAs of total microbes (nmol g^-1^ dry soil), bacteria (nmol g^-1^ dry soil), fungi (nmol g^-1^ dry soil), actinomycete (nmol g^-1^ dry soil), gram-positive bacteria (G+) (nmol g^-1^ dry soil), gram-negative bacteria (G-) (nmol g^-1^ dry soil), and values for the fungi:bacteria and G+:G- ratios, and the Shannon-Wiener diversity index, Margalef richness index, Pielou evenness index, and Simpson dominance index to experimental warming (W), plant treatments (P), and sampling dates (D).

Factor	MBC	MBN	MBC:MBN	Total microbial PLFAs	Bacterial PLFAs	Fungal PLFAs	Actinomycetic PLFAs	G+ PLFAs	G- PLFAs	Fungi:bacteria	G+:G-	Shannon-Wiener diversity index	Margalef richness index	Pielou evenness index	Simpson dominance index
D	**<0.001**	**<0.001**	0.997	**0.037**	0.132	0.613	**0.017**	**0.024**	**0.036**	**0.040**	**0.003**	0.384	0.179	**0.043**	0.074
D*P	**0.001**	**0.011**	**0.018**	0.585	0.815	0.324	0.463	0.823	0.826	0.075	0.935	**0.043**	**0.004**	0.581	**0.042**
D*W	0.667	**0.011**	0.134	**0.032**	0.138	**0.026**	0.055	0.151	0.260	0.089	**0.044**	0.509	0.938	0.261	0.462
D*P*W	**0.034**	**<0.001**	**0.025**	0.388	0.844	0.292	0.568	0.509	0.948	0.588	0.447	0.684	0.159	0.093	0.603
P	**0.002**	**0.020**	**0.004**	0.172	0.179	**0.009**	0.444	0.232	0.128	**0.003**	0.668	0.389	**0.006**	0.249	0.718
W	**0.001**	**0.004**	0.298	**0.024**	**0.012**	0.299	**0.009**	**0.011**	**0.003**	0.127	0.265	**0.007**	0.248	0.815	0.090
P*W	**0.004**	**0.050**	0.303	0.074	**0.046**	0.989	**0.030**	**0.032**	**0.036**	0.066	0.824	0.158	**0.050**	0.324	**0.007**

*P* values less than 0.05 are in bold.

## PLFAs of different components of soil microbial community

**Fig 3:**
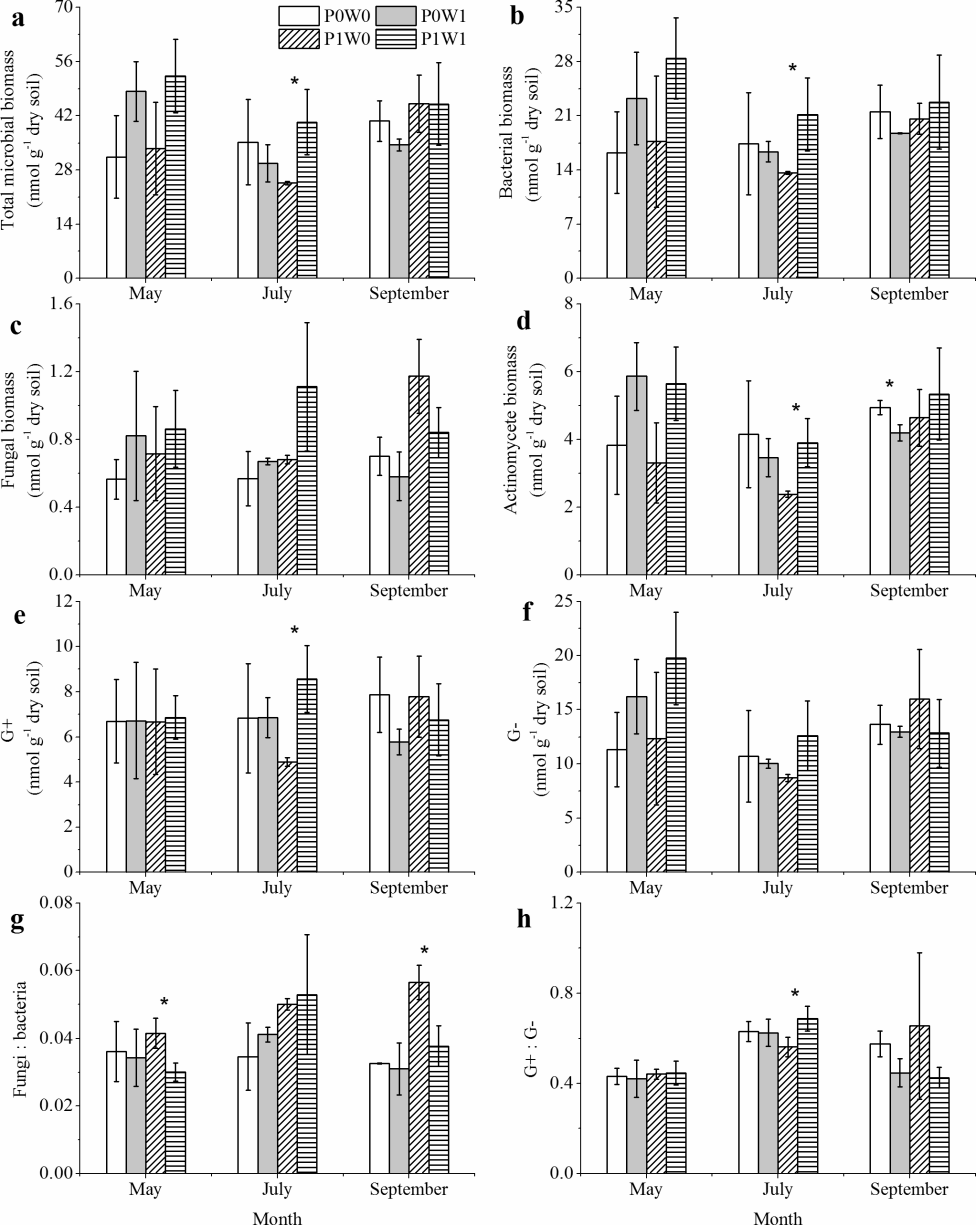
**Effects of experimental warming on the PLFAs of total microbes (a), bacteria (b), fungi (c), actinomycete (d), gram-positive bacteria (G+) (e), gram-negative bacteria (G-) (f), and the fungi:bacteria (g) and G+:G- (h) ratios during each sampling date, as indicated by the PLFA data.** P0W0, P0W1, P1W0, P1W1 refer to the treatments, indicating plant removal + unwarmed, plant removal + warmed, undisturbed + unwarmed, undisturbed + warmed, respectively. Asterisks indicate there were significant (*P* < 0.05) differences between the warmed and unwarmed plots at a given plant treatment (plant removal or undisturbed).

As indicated by the PLFA data, there were significant (*P* < 0.05) effects of experimental warming on the PLFAs of different components of the soil microbial community (Tables 1 and S1). Specifically, in the undisturbed plots, warming caused significant (*P* < 0.05) increases in the PLFAs of total microbes, bacteria, actinomycete and G+ and the ratio of G+ to G- (G+:G-) in July (Fig 3). Warming did not cause significant changes in the fungal PLFAs (Fig 3C), but it significantly (*P* < 0.05) decreased the ratio of fungi to bacteria (fungi:bacteria) in both May and September in the undisturbed plots (Fig 3G). In the plant-removal plots, however, warming had no significant effects on any microbial variable, except for September, when there was a significant decline in actinomycete PLFAs in the warmed plots (Fig 3D). Plant removal only significantly decreased fungal PLFAs and the fungi:bacteria ratio across the sampling dates (Fig 3C and Table 1). Like MBC and MBN, the PLFAs of total microbes, bacteria, actinomycete and G- in May were significantly higher than those in July and September (Fig 3, Tables 1 and S1). Furthermore, there were significant (*P* < 0.05) interactive effects of warming and plant removal on the PLFAs of total microbes, bacteria and actinomycete across the sampling dates (Table 1).

**Fig 4:**
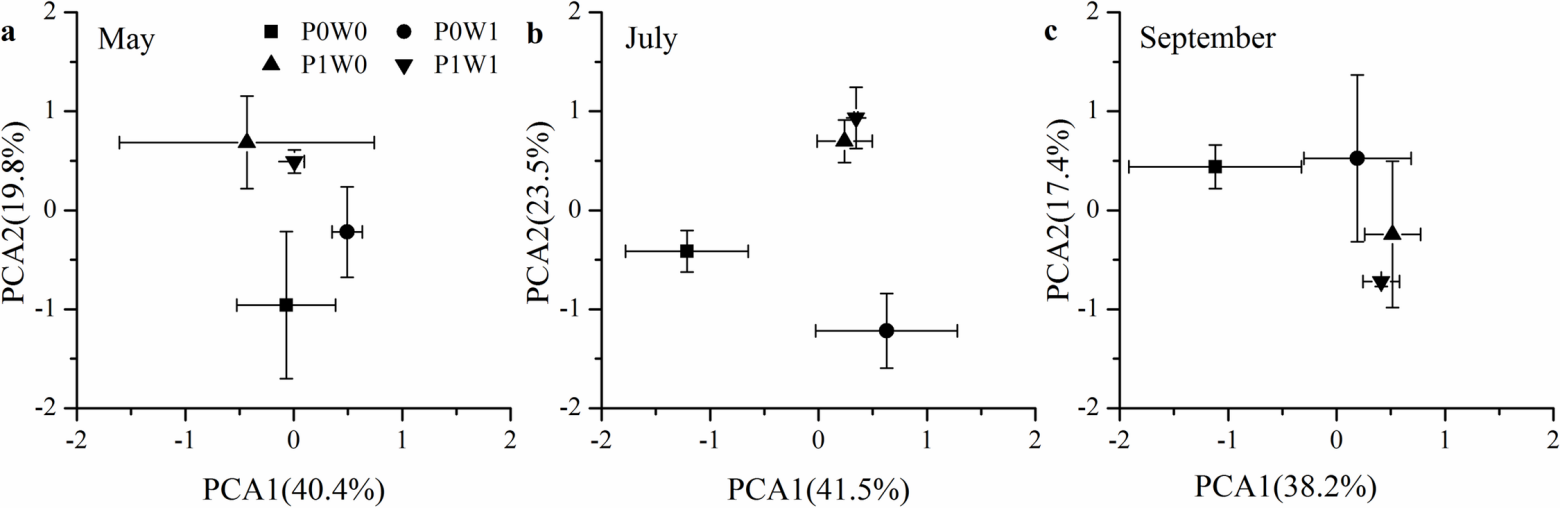
**Score plots of the two first components (PC) of the principal component analysis of the microbial PLFA content in May (a), July (b) and September (c).** P0W0, P0W1, P1W0, P1W1 refer to the treatments, indicating plant removal + unwarmed, plant removal + warmed, undisturbed + unwarmed, undisturbed + warmed, respectively.

To evaluate the changes in the soil microbial community structure, individual PLFA concentrations were used for PCAs within the plots of each sampling date. The PLFA variance explained by the two first principal components (PCA1 and PCA2) were 60.2%, 65.0% and 55.6% in May, July and September, respectively (Fig 4). In particular, the microbial community structure in the plant-removal plots was significantly (*P* < 0.05) different from that in the undisturbed plots in July (Fig 4B). However, no significant difference in the microbial community structure was detected in May and September, regardless of warming and plant treatments (Fig 4A–4C).

### Soil microbial PLFA diversity indices

**Fig 5:**
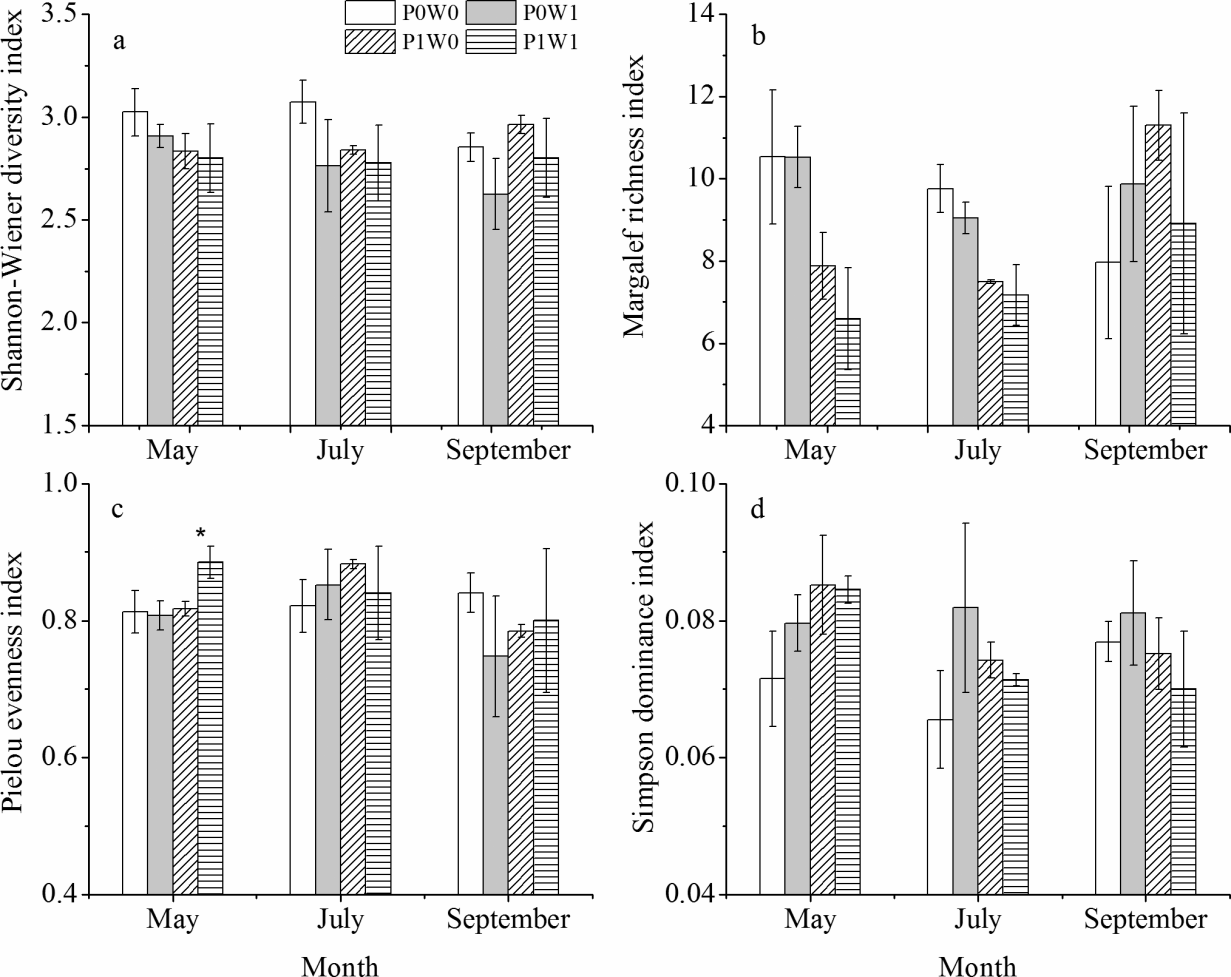
**Effects of warming on microbial PLFA diversity (a), richness (b), evenness (c), and dominance (d) at each sampling date.** P0W0, P0W1, P1W0, P1W1 refer to the treatments, indicating plant removal + unwarmed, plant removal + warmed, undisturbed + unwarmed, undisturbed + warmed, respectively. Asterisks indicate significant differences (* *P* < 0.05) between the warmed plots and the unwarmed plots at a given plant treatment (plant removal or undisturbed).

The repeated measures ANOVA showed that experimental warming did not significantly influence the richness, evenness and dominance indices of the soil microbial PLFAs over the three sampling dates (Fig 5, Tables 1 and S1), except for the undisturbed plots, in which warming significantly increased the Pielou evenness index in May (Fig 5C). Experimental warming significantly influenced the Shannon-Wiener diversity index, with lower values in the warmed plots than the unwarmed plots (Fig 5A, Table 1), while plant removal had a significant (*P* < 0.05) effect on the richness index. In addition, significant (*P* < 0.05) interactive effects of warming and plant removal on the richness and dominance indices were observed (Table 1).

## Discussion

### Microclimate as affected by warming and plant removal

In our study, OTCs increased the soil and air temperature by 0.53–1.64°C throughout the growing season in the alpine scrub ecosystem (Fig 1A and 1B), which was comparable with the ranges observed in similar alpine regions on the Qinghai–Tibetan Plateau that were studied using the OTC method [32]. However, the magnitudes of soil and air warming differed depending on the plant treatments. The increase in soil temperature in the undisturbed plots was higher than that in the plant-removal plots (Fig 1A). This was mainly due to the presence of shrub and grass contributing to the retention of heat fluxes in the soil produced by the passive OTC warming [33]. A strong positive correlation between plant biomass and soil warming was also observed in the high-latitude ecosystem [34]. Differences in the degree of other abiotic and biotic factors (e.g., wind speed and soil moisture) between the undisturbed and plant-removal plots might also partly explain the variability of the OTC effect on soil temperature [35]. For air temperature, lower increases in the undisturbed plot temperatures than the plant-removal plot temperatures were found in our study (Fig 1B). The increase in the transpiration of the aboveground part of shrubs and grasses absorbed a large amount of heat from the surroundings, which may alleviate the OTC effect on air temperature [36]. The canopy of shrubs and grasses intercept solar radiation, which could also reduce the heat flux and influence the variability of air temperature in the undisturbed plots [37]. Soil moisture was significantly decreased by the OTCs. In spite of the lower increase in soil temperature, the degree of the decrease in soil moisture in the undisturbed plots was higher than that in the plant-removal plots (Fig 1C). Our results were inconsistent with Wan et al (2002), who reported that warming significantly decreased soil moisture in the clipped plots, but not in the unclipped plots, of a tallgrass prairie [33]. In addition, an insignificant OTC effect on soil moisture was detected in an alpine meadow and an alpine shrubland due to soil moisture recharging through high amounts of precipitation [35]. In our study, the higher decrease in soil moisture in the undisturbed plots compared to the plant-removal plots might be attributed to the increase in the uptake, utilization and transpiration of soil moisture through the shrubs and grasses in the OTCs.

### Soil MBC and MBN as affected by warming and plant removal

Soil MBC and MBN are two important components in terrestrial ecosystem C and N cycling [38]. Soil MBC and MBN respond sensitively to changes in soil temperature and have been proposed to be important indicators for the prediction of potential impacts of climatic warming on soils [39]. In our study, experimental warming significantly influenced the soil MBC and MBN, but the warming effects differed among the sampling dates and plant treatments (Fig 2, S1 Table). In the undisturbed plots, experimental warming significantly increased the soil MBC in May and soil MBC and MBN in July. The results of the repeated measures ANOVA also showed that warming, plant treatments and the interactions between them significantly affected soil MBC and MBN (Table 1). More available C and N sources provided for soil microbes by greater amounts of root exudates produced by plant roots [40] and increases in plant litter decomposition and fine root turnover in warmed plots [14] could be responsible for the increases in soil MBC and MBN in this period. These results imply that warming-induced promotion of plant growth could potentially increase soil MBC and MBN in alpine regions [41]. Our results were similar with the results of a meta-analysis on the Qinghai–Tibetan Plateau, which reported that experimental warming significantly increased soil MBC and MBN by 14.3% and 20.1%, respectively. Experimental warming significantly increased soil MBN, slightly increased soil MBC and thus significantly decreased soil MBC:MBN in the plant-removal plots in May (Fig 2), mainly due to increases in the N mineralization rate and the decomposition of the remaining fine root litter caused by warming in this period [42,43]. However, no significant warming effect on the soil MBC and MBN was observed in the plant-removal plots in July and in both treatment plots in September. On one hand, a warming-induced decrease in the soil water content (Fig 1C) could partly account for the insignificant warming effect on the soil microbial biomass in our study. Our findings were in line with a study conducted in an alpine meadow on the Tibetan Plateau, which suggested that the absence of warming effects on soil microbial biomass might be related to decreases in soil water contents caused by warming [44]. On the other hand, fresh aboveground litter and root exudates were excluded in the plant-removal plots by removing all the aboveground biomass of shrub and grass species. This could cause nutrient limitations on soil microbes and would thereby counteract the positive effects of warming on soil microbial biomass. In addition, there were significant changes in the soil MBC and MBN among the three sampling dates, with higher values in May and lower values in July and September, regardless of warming and plant removal (Fig 2A and 2B), which suggests that the soil microbial community structure significantly changed over sampling dates due to the seasonal changes in soil temperature in the alpine scrub ecosystems on the Qinghai–Tibetan Plateau. The significant decreases in the PLFAs of actinomycete and G+ in July and September might partly explain the changes in the soil microbial community structure (Figs 3 and 4, S1 Table), which were indicated by seasonal changes in the soil MBC and MBN. Moreover, plant removal and the interactions of warming and plant removal had significant effects on soil MBC, MBN and MBC:MBN (Table 1). These results agreed with our hypothesis (2), indicating that the effects of experimental warming on soil microbial biomass can be modified by plant removal in the alpine scrub ecosystems on the Qinghai–Tibetan Plateau.

### Soil microbial community structure as affected by warming and plant removal

Climatic warming has the potential to alter soil microbial community structure, which could influence the function of terrestrial ecosystems. Alpine scrub ecosystems on the Qinghai–Tibetan Plateau are more ecologically sensitive to warming because all their biological activities are adapted to low temperatures [15]. In our study, warming did cause a significant increase in the total PLFAs in the undisturbed plots in May, mainly due to increases in the PLFAs of bacteria and actinomycete rather than increases in fungal PLFAs (Fig 3 and Table 1). The warming effects on the soil microbial community structure varied among different taxa [45], mainly because certain microbes with special adaptive traits might be more resistant to increases in soil temperature than others [46]. Warming did cause significant increases in the abundances of G+ and actinomycete, which was a result also reported by Frey et al (2008) in the Harvard Experimental Forest [47]. In an alpine meadow on the Qinghai–Tibetan Plateau, warming led to increases in the relative abundances of some bacterial groups but decreases in those of other bacterial groups, as indicated by a soil transplant experiment. These results suggest that the responses of soil microbial community structures to warming significantly varies among the different taxa of soil microbes in alpine scrub ecosystems, which supports our hypothesis (1). In addition, warming-induced increases in plant growth would contribute significantly to shaping the soil microbial community structure [45]. Compared with the abundances of fungi, experimental warming significantly increased the relative abundances of bacteria groups in May and September in the undisturbed plots, as suggested by significant decreases in the fungi:bacteria ratio (Fig 3). The increases in plant products (i.e., root exudates and fresh litter) might directly benefit actinomycete and bacteria groups rather than fungi groups in these alpine ecosystems. Another reason for the increase in bacterial abundances is that bacteria groups dominated the soil microbial community in our study site (Fig 3B), and warming likely enhanced the bacterial dominance [16]. In addition, the increases in plant growth in the undisturbed plots caused by warming could strengthen the competition for soil labile nutrients between the plants and soil microbes, which might indirectly increase the relative abundances of oligotrophic taxa, such as actinomycete groups [25], especially in the mid-growing season. Furthermore, warming caused a significant shift in the soil microbial community towards actinomycete and G+ in the undisturbed plots (Fig 3D and 3E), which suggests that actinomycete and G+ groups are more sensitive to experimental warming and might be good indicators for the prediction of potential changes in soil microbial community structures in responding to climatic warming in alpine scrub ecosystems. Similar to its effect on soil MBC, experimental warming had no significant effect on any variable of the soil microbial community component in the plant-removal plots, which suggests that plant removal reduces the temperature sensitivity of soil microbial communities in alpine regions, possibly due to the changes in soil microenvironmental conditions, such as decreases in soil moisture and nutrients [44]. Although plant removal alone did not significantly influence the PLFAs of bacteria and actinomycete, warming and plant removal had significant interactive effects on these groups.

Previous studies have reported that the fungal community may be particularly sensitive to climatic warming in alpine regions due to the removal of temperature limitation in the plant-soil system [48]. However, in our study, experimental warming did not incur significant changes in fungal PLFAs in the plant-removal and undisturbed plots, which indicates that fungi groups were less sensitive in response to warming than other groups. In alpine ecosystems, fungi groups have a certain ability to tolerate and adapt to soil warming [49]. The decreases in soil moisture in the warmed plots were insufficient to alter the fungal community because fungal hyphal networks expand when soil water is limited to access more moisture and nutrients [50]. These results suggest that fungi groups might play an important role in moderating the soil microbial community responses to short-term warming in alpine scrub ecosystems. However, inconsistent results have also been observed in the similar alpine ecosystems. Studies have shown that experimental warming significantly decreased fungal abundances in a subalpine coniferous forest as a result of decreases in soil moisture and nutrients [6] and increased fungal abundances at an alpine tree line in the Central Alps due to increases in fine root biomass and extractable NH_4_^+^ [48]. Our results also suggest that plant removal significantly reduced fungal PLFAs, regardless of warming (Fig 3C, Table 1), and the soil microbial community structure significantly differed between the plant-removal and undisturbed plots in July (Fig 4), which implies that plant communities might play important roles in shaping soil microbial communities through a significant shift in fungi groups. Warming-induced changes in plant growth, plant community composition and soil nutrients might have stronger effects on fungi groups than other taxa in alpine ecosystems [16].

### Soil microbial PLFA diversity as affected by warming and plant removal

It is well documented that soil temperature plays a more prominent role in shaping variations in soil microbial diversity than other proposed environmental drivers [51]. In this study, indices of microbial PLFA diversity, richness, evenness, and dominance were calculated for the microbial PLFAs at different dates. In particular, the Shannon-Wiener diversity index gave weight to the rarer taxa while the Simpson dominance index gave weight to the more abundant taxa [52]. In our study, we found a significant decrease in the diversity index of the warmed plots (Fig 5A, Table 1), which indicates that warming could significantly reduce the abundance of rare taxa. Plant removal also significantly influenced the Margalef richness index (Table 1). However, the combination of warming and plant removal tended to have strong effects on the abundant taxa rather than the rare taxa due to significant interactive effects on the Simpson dominance index and Margalef richness index (Table 1). Therefore, our results suggest that warming effects on the diversity of the soil microbial community are regulated by the plant community in alpine scrub ecosystems [53].

## Conclusions

The present study elucidated the effects of warming, plant treatments and the interactions between them on the soil microbial communities of the alpine scrub ecosystems on the eastern Qinghai–Tibetan plateau. Our results revealed that (1) the combination of experimental warming and plant removal significantly influenced the soil MBC and MBN in the alpine scrub ecosystems; (2) warming significantly increased the abundances of actinomycete and G+ in the undisturbed plots during the mid-growing season, while fungi groups were significantly affected by plant removal rather than warming; and (3) only experimental warming significantly reduced the abundance of rare taxa; however, the combination of warming and plant removal tended to have strong effects on the abundant taxa. These results suggest that the crucial roles of plant communities need to be preferentially considered when we assess the effects of climatic warming on soil microbial processes in alpine ecosystems. Our results will expedite the full understanding of C and nutrient cycling under climatic warming as affected by soil microbes in alpine scrub ecosystems.

## Supporting information

S1 Table Results of the mixed model showing the *P* values for the effects of experimental warming (W), plant treatments (P) and their interactions (P*W) on soil microbial variables. (DOC)

S1 Data Data for PONE-D-17-15877 (including microbial variables, temperature and soil moisture parameters). (XLS)
