# Supplementary material for: Plants regulate the effects of experimental warming on the soil microbial community in an alpine scrub ecosystem
Source: PLoS One. 2018 Apr 18;13(4):e0195079. doi: 10.1371/journal.pone.0195079 (PMC5905891; doi:10.1371/journal.pone.0195079)
Supplement: S1 Table — (DOC) [file pone.0195079.s001.doc]

**S1 Table. Results of the mixed model showing the *P* values for the effects of experimental warming (W), plant treatments (P) and their interactions (P*W) on soil microbial variables.**

| Sampling date | Factors | MBC | MBN | MBC:MBN | Total microbial PLFAs | Bacterial PLFAs | Fungal PLFAs | Actinomycetic PLFAs | G+ PLFAs | G- PLFAs | Fungi:bacteria | G+:G- | Shannon-Wiener diversity index | Margalef richness index | Pielou evenness index | Simpson dominance index |
| --- | --- | --- | --- | --- | --- | --- | --- | --- | --- | --- | --- | --- | --- | --- | --- | --- |
| May | Block | 0.426 | 0.567 | 0.427 | 0.726 | 0.596 | 0.745 | 0.981 | 0.965 | 0.823 | 0.409 | 0.404 | 0.075 | 0.133 | 0.062 | 1.000 |
|  | W | 0.062 | **0.003** | **0.005** | **0.028** | **0.040** | 0.454 | **0.023** | 0.084 | 0.056 | 0.164 | 0.829 | 0.357 | 0.356 | **0.046** | 0.592 |
|  | P | **0.008** | **0.004** | **0.003** | 0.636 | 0.499 | 0.402 | 0.631 | 0.376 | 0.500 | 0.998 | 0.479 | 0.103 | **0.002** | **0.028** | 0.308 |
|  | W*P | 0.051 | **0.014** | 0.125 | 0.887 | 0.858 | 0.409 | 0.838 | 0.619 | 0.794 | 0.613 | 0.608 | 0.594 | 0.361 | **0.028** | 0.592 |
| July | Block | 0.053 | 0.136 | 0.149 | 0.135 | 0.391 | 0.228 | 0.255 | 0.184 | 0.986 | 0.536 | 0.079 | 0.364 | 0.181 | 0.306 | 1.000 |
|  | W | **0.010** | 0.127 | 0.233 | **0.033** | **0.043** | 0.276 | 0.126 | **0.049** | 0.251 | 0.567 | 0.066 | **0.039** | 0.124 | 0.225 | 0.097 |
|  | P | **0.004** | 0.449 | **0.015** | 0.999 | 0.802 | **0.020** | 0.306 | 0.855 | 0.808 | **0.036** | 0.716 | 0.286 | 0.075 | 0.409 | 0.760 |
|  | W*P | **0.005** | **0.007** | 0.077 | 0.298 | 0.227 | 0.128 | 0.495 | 0.146 | 0.313 | 0.355 | 0.156 | 0.100 | 0.092 | 0.864 | 0.387 |
| September | Block | 0.252 | 0.061 | 0.758 | 0.837 | 0.798 | 0.060 | 0.207 | 0.792 | 0.984 | 1.000 | 0.816 | 0.677 | 0.663 | 0.303 | 0.055 |
|  | W | 0.115 | 0.877 | 0.378 | 0.506 | 0.450 | 0.310 | 0.185 | 0.547 | 0.472 | 0.224 | 0.739 | **0.047** | 0.852 | 0.255 | 0.776 |
|  | P | 0.683 | 0.734 | 0.774 | 0.137 | 0.340 | **0.013** | 0.396 | 0.756 | 0.151 | 0.028 | 0.172 | 0.193 | 0.363 | 0.488 | 0.414 |
|  | W*P | 0.844 | 0.299 | 0.311 | 0.492 | 0.340 | 0.063 | 0.964 | 0.121 | 0.988 | 0.224 | **0.034** | 0.356 | 0.131 | 0.179 | 0.414 |

*P* values less than 0.05 are in bold.
